# Qualitative assessment of evidence-informed adolescent mental health policymaking in India: insights from project SAMA

**DOI:** 10.1186/s12961-024-01184-w

**Published:** 2024-09-18

**Authors:** Alice Ivory, Mutharaju Arelingaiah, Navaneetham Janardhana, Poornima Bhola, Siobhan Hugh-Jones, Tolib Mirzoev

**Affiliations:** 1 Department of Global Health and Development, London School of Hygiene and Tropical Medicine, London, United Kingdom; 2 Department of Psychiatric Social Work, National Institue of Mental Health and Neurosciences, Bangalore, India; 3 Department of Clinical Psychology, National Institute of Mental Health and Neurosciences, Bangalore, India; 4 School of Psychology, University of Leeds, Leeds, United Kingdom

**Keywords:** Adolescent mental health, Health policy, Evidence-informed policy, India, Asia low- and middle-income countries

## Abstract

**Background:**

The importance of evidence-informed health policymaking is widely recognized. However, many low- and middle-income countries lack evidence-informed mental health policies due to insufficient data, stigma or lack of resources. Various policies address adolescent mental health in India, but published knowledge on their evidence-informed nature is limited. In this paper, we report results of our analysis of the role of evidence in adolescent mental health policymaking in India.

**Methods:**

This paper reports findings from the document analysis of key policy documentation (*n* = 10) and in-depth interviews with policy actors including policymakers, researchers, practitioners and intermediaries (*n* = 13). Framework analysis was used, informed by the components of a conceptual framework adapted from the literature: actors, policy and evidence processes, nature of evidence itself and contextual influences.

**Results:**

Results show that adolescent mental health policies in India were generally evidence-informed, with more key evidence becoming generally available from 2010 onwards. Both formal and informal evidence informed mental health policies, particularly agenda-setting and policy development. Mental health policymaking in India is deemed important yet relatively neglected due to competing policy priorities and structural barriers such as stigma. Use of evidence in mental health policymaking reflected differing values, interests, relative powers and ideologies of policy actors. Involvement of government officials in evidence generation often resulted in successful evidence uptake in policy decisions. Policy actors often favoured formal and quantitative evidence, with a tendency to accept global evidence that aligns with personal values.

**Conclusions:**

There is a need to ensure a balanced and complementary combination of formal and informal evidence for policy decisions. Evidence generation, dissemination and use for policy processes should recognize evidence preferences by key stakeholders, while prioritizing locally available evidence where possible. To help this, a balanced involvement of policy actors can ensure complementary perspectives in evidence production and policy agendas. This continued generation and promotion of evidence can also help reduce societal stigma around mental health and promote mental health as a key policy priority.

## Introduction

There is growing recognition of the importance of evidence-informed health policy decisions to inform responses to public health priorities [1–11]. Systematic use of evidence in policymaking nowadays is seen as key to efficient use of public expenditure to promote population health [12–14].

Mental health policy is increasingly recognized as a global development priority [13, 15]. In low- and middle-income countries (LMICs), up to 85% of people with mental health conditions are untreated [16]. One reason for this treatment gap is the challenge of translating evidence into policymaking, which in the context of LMICs and mental health can be exacerbated due to surrounding stigma, limited political attention and a lack of adequate infrastructure [17–19]. This can result in the absence of, or constrained, implementation of mental health policies [17–20]. These barriers mean not only that many LMICs lack a stand-alone mental health policy [13, 21] but also that existing policies may not be evidence informed due to insufficient data or competing priorities on the political agenda which demand more research resource [19, 22].

Frameworks have been developed for the use of evidence-based health policy agenda-setting in LMICs [6, 13], but their use is limited, especially in regard to mental health policy. Some frameworks which respond to this gap specifically focus on research-policy interrelationships in mental health policymaking in LMICs [16]. However, literature tells us that evidence comes in various forms including formal (e.g. scientific research, national surveys) and informal (e.g. personal experiences, expert opinions) [2, 4–6, 13, 23], thus highlighting the importance of all types of evidence, especially regarding potentially sensitive or stigmatized issues such as mental health.

Adolescent mental health is a priority in India, which has the largest number of adolescents globally (243 million). It is estimated that one in five school-going adolescents live with anxiety, stress and/or depression [24]. While policies exist which address mental health and adolescent health in India, little is known about whether any evidence informs development and implementation of these policies.

This paper aims to bridge this gap by reporting results of analyses of the role of evidence in adolescent mental health policymaking in India, using an adapted conceptual framework to the Indian context [5]. We hope this article will be of interest and relevance to decision makers, researchers and intermediaries who are interested in advancing their understanding and improving the role of evidence in mental health policymaking in India and beyond.

## Methods

We report results from a qualitative policy analysis, which examined the role of evidence in mental health policymaking in India, from a component of a wider SAMA project [25].

### Analytical framework

The following theorization of the role of evidence in mental health policymaking provided analytical framing and informed the structure of the data collection and analysis.

A multitude of frameworks exist for health policy analysis which have been extensively applied to identify and understand complex interactions that shape the development and implementation of national policies. The most widely used framework is the ‘policy triangle’ [25]. The triangle identifies four interrelated components: processes (how policies are made), actors (by whom policies are made), context (wider issues affecting policies) and contents (of the policy). The Stages Heuristic Model [26] explains four iterative stages of policy processes: agenda setting (recognizing the problem and getting the issue onto the policy agenda), policy development (developing the policy response to the issue), policy implementation (implementing the policy change) and policy evaluation (monitoring and evaluation of the implemented policy to assess whether it has achieved the desired effect). Frameworks also exist that help understand and explain involvement of different actors in policymaking [25, 27] and wider contextual environment [2, 5, 28–31].

Further theorizations of evidence-informed policymaking have also been reported, essentially proposing different models for evidence-informed decision-making, identifying key actors involved in the process (researchers, decision makers and intermediaries) including their characteristics and roles, proposing different taxonomies of evidence and increasingly applying this knowledge to advance the understanding of evidence-informed mental health policymaking [13, 16], though with less focus on adolescent mental health policies.

**Fig. 1:**
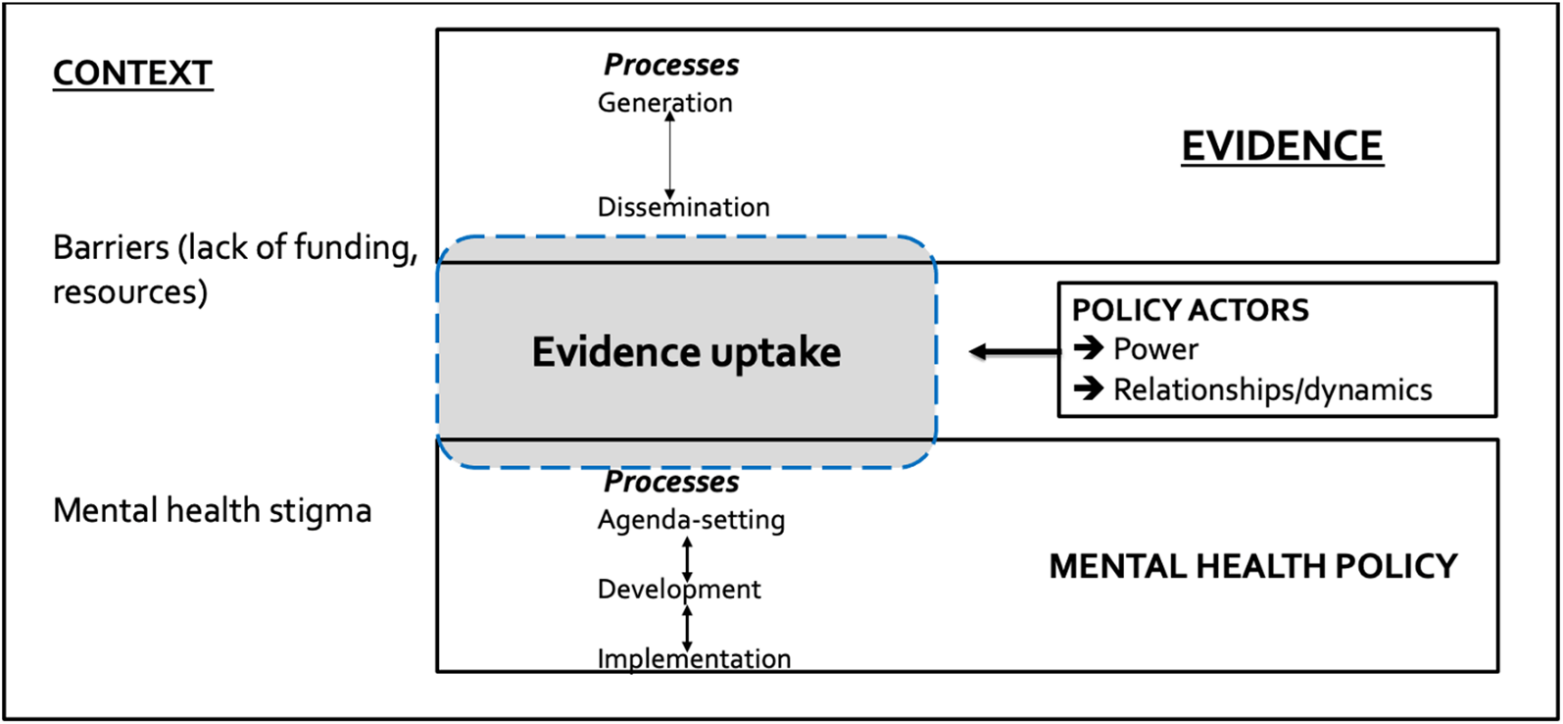
Conceptual framework for understanding role of evidence in mental health policymaking (Mirzoev et al. 2017)

We build on, and consolidate, these bodies of knowledge, in adapting a conceptual framework from Mirzoev et al. (2017) to understand evidence-informed policymaking in the context of adolescent mental health (Fig. 1).

This framework highlights the complex interrelationship between two overlapping processes: evidence processes (generation, i.e. when research is conducted or when experiences are accumulated; dissemination, i.e. when evidence is shared with policy actors; and uptake, i.e. when evidence informs policy decisions) and policy processes (agenda-setting, policy development and policy implementation). Evidence generation, dissemination and uptake can all occur within a single stage of the policy process (such as agenda setting) or can cut across multiple stages.

Different policy actors may engage in either evidence processes or policy processes, or both: policymakers (e.g. government officials, chief scientific advisors, politicians and committee members), intermediaries (organizations or individuals who work between policymakers and service providers, e.g. government advisors) and evidence producers (e.g. researchers, NGOs, think tanks, and academic institutions). These actors determine the relationship between the evidence processes and policy processes and, consequently, a degree of evidence use within policy processes. Actors can have different perceptions of what constitutes appropriate or robust evidence for policymaking, often influencing which policy decisions are informed by which evidence. These actors have their own and sometimes differing, values, interests, agendas and relative powers which influence decisions regarding evidence uptake [6, 25, 32].

Lastly, the framework delineates how engagements of policy actors with evidence and policy processes occur within and are influenced by various contextual factors which often cut across individual, organizational and system levels [5, 25, 33]. Two specific contextual influences reflect the nature of adolescent mental health policymaking in India in our adapted framework: structural barriers and mental health stigma.

### Data collection and analysis

Two methods were used to collect data: (1) desk review of relevant policies and guidelines (*n* = 10) and (2) in-depth interviews (*n* = 13).

We identified and reviewed 10 national and state-level policies, presented across Tables 1 and 2 alongside their objectives. Our initial analysis primarily focused on four mental health policies due to their relevance to adolescent mental health issues (Table 1).

**Table 1:** Four key policies analysed in the study

No	Policy document	Policy focus/objectives	Corresponding evidence
1	National Mental Health Policy (2014)	Within a rights-based framework, the policy aims to promote mental health and de-stigmatization and provide accessible quality health and social care to all individuals	Drew upon national, state-level reports and survey-data and WHO frameworks/reports. Expert discussions and consultations were also used in agenda setting and policy development
2	Ayushman Bharat (2018)	Recognizing schools as useful platforms, the ‘School Health Programme’ under Ayushman Bharat aims to strengthen health promotion and disease prevention interventions. Aims to strengthen the implementation of existing programmes including RBSK (2013) and RKSK (2014) (Table 2)	Policy development was informed by the national and state-level reports and survey-data, as well as WHO frameworks and reports. Expert discussions and consultations also an important part of agenda-setting and policy development process
3	National Education Policy (2020)	The policy seeks to increase public investment in education and proposes the revision of all aspects of the education structure, aligned with the goals of twenty-first century education. The policy holds India’s tradition, culture and value system close to its core	Policy was formulated through the uptake of extensive expert consultations and public feedback via Twitter and Facebook. This was due to a lack of evaluations data since the implementation of the last education policy
4	National Suicide Prevention Strategy (2022)	Strategy aims to enhance the capacity of health services to provide suicide prevention services, develop support for suicide prevention and de-stigmatization of suicidal behaviours	Policy largely based upon the National Crime Records Bureau, resulting from a lack of suicide data in India. Existing policies such as the National Mental Health Policy, global WHO reports and international studies were also used. Expert discussions and consultations informed policy agenda-setting and development

**Table 2:** Additional adolescent mental health policies reviewed

No	Policy document	Policy focus/objectives
5	A Strategic Approach to Reproductive, Maternal, Newborn, Child and Adolescent Helath (RMNCH + A) in India (2013)	Strategy aims to link maternal and child health to reproductive health and other components (e.g. family planning, adolescent health, human immunodeficiency virus (HIV))
6	Rashtriya Bal Swasthya Karyakram (RKSK) Operational Guidelines (2013)	Initiative focuses on early identification and intervention to improve survival outcomes amongst children 18 years old and under
7	National Adolescent Health Strategy Handbook (RKSK) (2014)	Strategy aims to increase availability and access to information about adolescent health and quality counselling and health services for adolescents
8	Rashtriya Kishor Swasthya Karyakram (RKSK) Operational Framework (2014)	Operational Framework is a component of the National Adolescent Health Strategy and assists states in implementation of the strategy. Framework provides guidance, reports, and operationalization
9	Health Vision Document for Karnataka (Disability Inclusion in Health and Family Welfare) (2021)	Aims to provide continuous high-quality comprehensive health care and rehabilitation services integrated with primary, secondary, tertiary prevention–rehabilitation services for people with disabilities
10	National Council of Educational Research and Training (NCERT) Guidelines (2022)	Module designed for teachers to raise awareness and enhance sensitivity towards mental health issues and concerns in schools

We also drew on other existing mental health policies (*n* = 6) from 2006 to 2022 to identify the interconnecting ways that evidence generation and uptake can inform future policy agenda-setting and development (Table 2).

A semi-structured template was used to capture key information from each document in a standardized format, and included information about the document, its contents and evidence cited, policy actors and further information on the role of evidence. The document review informed a stakeholder mapping to identify informants for in-depth interviews. Document review and stakeholder mapping were conducted over the course of initial two months.

A total of 13 in-depth interviews were conducted via Zoom and in-person. Ten interview respondents were directly involved in the evidence generation, development or implementation of the four identified policies and three respondents have extensive history in mental health policymaking but not specifically these four policies. Table 3 presents the role and linked organization of interview participants.

**Table 3:** Participant characteristics

Participant category	*N*	Organizations
Policymaker	4	Ministry of Health and Family Welfare (MoHFW); Ministry of Education (MoE); Govt of Karnataka
Researcher	6	NIMHANS; Ramaiah University; Chanakya University; UNICEF
Intermediary (regional advisor)	1	Bangalore University
Practitioner (psychiatrist, school counsellor)	2	NIMHANS; local schools
Total	13	

Interviews were informed by a semi-structured question guide, which reflected our conceptual framework: evidence processes, policy processes, policy actors and policy context. An initial question guide was pilot-tested within the research team and revised for the data collection. Interviews lasted between 45 min and 1 h. The question guide was adjusted to the roles of specific interviewees. Interviews were transcribed verbatim for analysis.

All data from the documents and interviews was subjected to framework analysis [34, 35], which involves stages of (1) familiarization, (2) identifying a thematic framework, (3) indexing, (4) charting and (5) mapping and interpretation. Data analysis was conducted alongside ongoing data collection to inform subsequent steps; for example, the document review informed an initial list of informants and results from initial interviews informed specific themes that were probed during subsequent interviews. Findings were continuously triangulated between document reviews and interviews.

### Ethics

The project complied with high ethical standards for collaborative global health research. Ethics approvals were obtained from the ethics committees from the National Institute for Mental Health and Neurosciences, the London School of Hygiene and Tropical Medicine and the University of Leeds.

## Results

We report results following the components of our analytical framework (Fig. 1). First, we will describe the policy processes following the Stages Heuristic Model of each policy, followed by the uptake of evidence across all policies. We present findings using visualizations of critical timelines. We then discuss the context and nature of mental health policymaking in India and the ways stakeholder power and dynamics influenced the uptake or rejection of evidence. Finally, we will highlight the barriers, facilitators and opportunities for evidence-informed mental health policymaking.

### Mental health policymaking in India

All four policies went through stages of agenda setting, development and implementation, though with differences reflecting the nature of each policy issue (Tables 1 and 2, 3) Agenda-setting generally involved advocacy and used previous policy iterations as opportunity for evidence generation. For example, the National Mental Health Policy and Ayushman Bharat sought new evidence based on emerging issues or gaps that previous policies or guidelines did not address. An interviewee with a member of the technical committee for the Suicide Prevention Strategy stated that the policy was put onto the agenda due to increasing public and political concern around the small amount of evidence that was available, despite alarmingly high rates of suicide and suicide attempts. Policy development was typically via participatory approaches such as technical committees and individual expert inputs. Alongside professional inputs, the National Education Policy also used public feedback through social media in efforts to secure more comprehensive consultation.

The implementation of each policy differed due to policy mandates at different levels (e.g. education, disability, and health are state subjects), involvement of stakeholders and the degree of integration of health services at district and/or state level. As the National Education Policy and the Suicide Prevention Strategy have both been introduced recently, their implementation is nascent. However, the National Mental Health Policy and Ayushman Bharat have faced several barriers during their implementation and uptake of their programmes [36] (Table 4).

**Table 4:** Policy processes of four key documents

	Policy			
	National Mental Health Policy (2014)	Ayushman Bharat (2018)	National Education Policy (2020)	National Suicide Prevention Strategy (2022)
Agenda setting	Follows the broad agendas of previous National Mental Health Policy (1982). In the World Health Assembly (2012) India committed to a new mental health policy, addressing the need for a comprehensive and coordinated response at the community level	Partnership between the Ministry of Health and Family Welfare (MoHFW) and Ministry of Education (MoE) with a focus on children’s health. This School and Wellness Programme built on the initial component of the operational guidelines and after the National Health Policy (2017) recommended strengthening the delivery of primary health care	First education policy since 1986. The Government of India (GoI) initiated the process based on the changing Indian child and youth population and their mission with regard to high-quality education	The MoHFW announced the policy based on rising suicide rates in India. The first policy in India to make suicide prevention a public health priority and ensures to remain in-line with India’s cultural and social milieu
Development	Government of India (GoI) constituted a policy group to recommend a new mental health policy for the country. Consultations and discussions were held in accordance with the mandate following which the group submitted recommendations	Other ministries (e.g. Ministry of Women and Child Development) were onboarded, as they were focusing on the health of young children in their own schemes. Consultations with subject experts followed	Aimed for an inclusive, participatory, and holistic consultation. Considered expert opinions, field experiences, research and stakeholder feedback and practices. An expert committee received feedback on a draft report in 2019 from government ministers and the public via Facebook and Twitter. Meeting of Central Advisory Board of Education was held and decision makers gave inputs. Draft Cabinet Note was circulated for inter-ministerial review during a second round of consultations	A technical committee was formulated, comprising stakeholders from various departments, ministries and sectors whose opinions and experiences informed the first draft. First draft of the document was deliberated by the Technical Committee Meeting and inputs were invited from the members of the Committee
Implementation	Provision for community based rehabilitation of persons with mental illness and delivery of mental health services through an integrated care model. Local implementation remains low as inadequate support from executory bodies, limited resources and links among services, scarcity of qualified staff at the community level and continued public mental health stigma (Gupta and Sagar, 2022)	The National Health Authority was established as an organization to administer the programme. Recommended holistic health care provision, including mental health, at primary health centres with collaboration with private sectors and NGOs. Various Indian states have declined to implement the programme as they either had their own state healthcare programmes or were in the process of establishing their own	Implementation is led by various bodies, i.e. Union and State governments, MoE, State Departments of Education, National Testing Agency, regulatory bodies of school and higher education, schools, and higher education institutions. In August 2021, the state of Karnataka became the first state to issue an order with regard to implementing the NEP. Full implementation is currently in progress and is yet to be evaluated	Strategy includes an implementation framework for various activities aimed at achieving objectives, targeting multilevel stakeholders. Strategy recognizes the crucial role of existing programmes to build upon or with. Full implementation is in progress due to the recent development of the strategy

### Uptake of evidence across policies

**Fig. 2:**
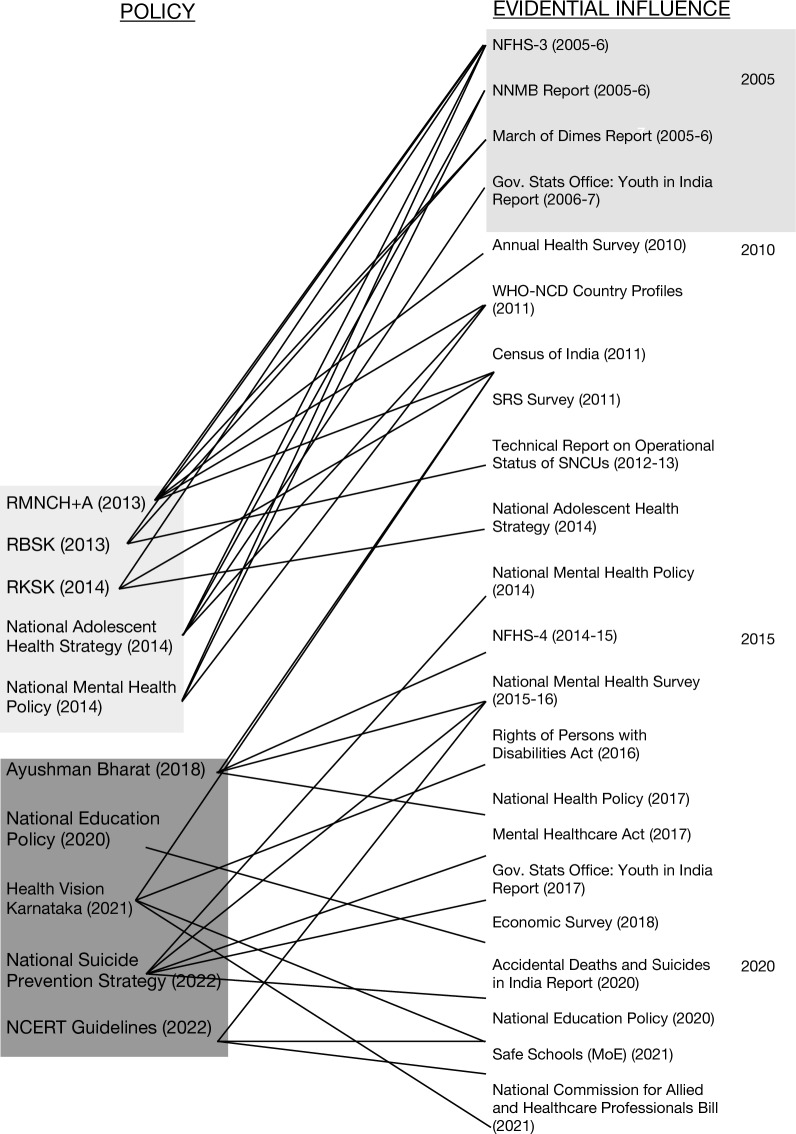
Timeline of critical evidential influences within mental health policymaking in India

Our analysis showed that between 2005 and 2010 there was little available evidence that informed Indian adolescent mental health policies, highlighted by grey shading in Fig. 2. However, an outburst of evidence occurred from 2010 onwards (Fig. 2), which informed mental health policies. There is a clear upwards trend in the amount of evidence from 2010 onwards, showing the link between evidence generation and policy development and implementation. The agenda was set in 2006 by the National Family Health Survey-3, National Nutrition Monitoring Bureau Report and March of Dimes Report, which informed the development of policies up to 6 years later. The implementation of three significant policies in 2014 (RKSK, National Adolescent Health Strategy and National Mental Health Policy) occurred just 2–3 years after an abundance of relevant evidence was published between 2010 and 2014. This upwards trend between evidence generation and policy implementation continued until 2022. The first five policies (RMNCH + A, RBSK, RKSK, National Adolescent Health Strategy and National Mental Health Policy) in Fig. 2 are shown in grey shading to highlight that the first ten key sources of evidential influence (NFHS-3 in 2005 to National Adolescent Health Strategy in 2014) were only used to inform these five policies. This is excluding the Health Vision Karnataka document that utilized the Census of India report 10 years after it was published in 2021. Similarly, the remaining five policies (Ayushman Bharat, National Education Policy, Health Vision Karnataka, National Suicide Prevention Strategy and NCERT Guidelines) are grouped together to reflect the ways in which a new generation of evidence (in this case from the National Mental Health Policy in 2014 to the National Commission for Allied and Healthcare Professionals Bill in 2022) is used to inform new iterations of policies from Ayushman Bharat in 2018 to NCERT Guidelines in 2022.

As Fig. 2 shows, at least one form of key evidence was used to inform each policy during agenda setting or development. Contents of some policies were also used as ‘evidence’ to inform further policies; for example, the National Adolescent Health Strategy was used to inform RKSK. Another example is that the NFHS-3 (2005) informed the National Mental Health Policy (2014), which in turn influenced agenda-setting for the National Suicide Prevention Strategy. This emphasizes the interconnecting ways in which evidence informs policy decisions and how evidence generation, uptake and dissemination is key for further health policy development and implementation (highlighted by grey shading of policies across the timeline).

A combination of both formal and informal evidence was used to inform these four national mental health policies. Document reviews, perhaps understandably, did not highlight the uptake of informal evidence (excluding expert committees, which were included in the development of all four policies). However, respondents noted in interviews that many policies were also informed through informal evidence such as personal experiences. Document reviews highlighted the uptake of formal evidence such as local and international research publications, census data and epidemiological surveys to inform all policies, viewed as the most ‘robust’ forms of evidence amongst all stakeholders during interviews. Interviews with a government official from MoE and a researcher suggested how critical evidence was for the National Education Policy (2020). Due to absence of evaluation data after the first Education Policy (1986), the New Education Policy was formulated through the uptake of extensive expert consultations and public feedback via Twitter and Facebook.

As Fig. 2 shows, majority of evidence utilized in both agenda setting and development stages of the policies are formal, quantitative, national-level reports. Document reviews suggested that this form of evidence was widely utilized, rarely sourcing qualitative evidence. However, policy processes would sometimes include the use of qualitative methodologies such as focus groups or discussions with community members and stakeholders, also generating further evidence for future policy iterations. Document reviews showed the use of WHO frameworks and international studies, used in the development of 6 of the 10 policies. These reports were all quantitative epidemiological studies. WHO frameworks were sometimes tailored to fit the Indian context or from countries with similar mental health prevalence. Similarly, as Fig. 2 shows, national-level surveys and reports were commonly used amongst all policies and were often perceived as the most robust forms of evidence during interviews, alongside global data. State-level surveys and reports were not featured much in the reviewed documents; however, interviews would sometimes draw on issues as ‘state-level issues’ that are often based on political priority or capacity per state and, thus, may differ between national-level policies. Similarly, although local research publications were used to inform five policies, it was more common for either international WHO data to be used or national-level epidemiological or survey data.

Although a lack of available evidence often reduces attention on the policy agenda, the National Suicide Prevention Strategy countered this. The lack of evidence was used as an opportunity to draw attention to neglected issues and drive the policy agenda and includes plans to generate further evidence via monitoring and evaluation of the strategy. Interestingly, our study interviews with researchers also emphasized the importance of advocacy during evidence generation and dissemination to enhance the credibility of evidence to decision makers and support evidence-informed policy development. An interview with one researcher described the uptake of advocacy briefs as an instrumental component during policy formation, which are submitted to the government so they are aware of policy needs.

### Contextual influences on role of evidence

We found that perceived societal stigma surrounding mental health in India can influence policy agenda and evidence generation and uptake. Our document analysis identified that all 10 policies either aimed or had subaims to promote mental health, prevent mental illness and ensure interventions or activities are in place to do so. However, interview data from two researchers suggested that competing priorities (e.g. non-communicable diseases) meant mental health issues receive less attention during agenda-setting and less funding and resources for policy implementation. As one researcher described, non-communicable diseases often get prioritized due to their ‘visibility’ and clear economic burden. Our analysis suggests that low political priority can also lead to limited funding and constrained evidence generation, as was seen in the National Suicide Prevention Strategy.

Although the Mental Healthcare Act (2017) recently decriminalized suicide and suicide attempts, there remains sensitivity and controversy surrounding suicidal behaviour. The development of the National Suicide Prevention Strategy was largely informed by the National Crime Records Bureau, which reports suicides as ‘accidental deaths and suicides’. An interview with a psychologist stated that many families may also not report suicide attempts or mental health disorders due to perceived shame or may report these cases as ‘accidents’. This can lead to a skewed representation of data and limited evidence to inform policy agenda, as one interviewee stated:

What I think was more predominantly highlighted was actually the lack of evidence. That was the concern people had…is this strategy going to really work in parts of the country where we don’t have the evidence… (Mental health practitioner).

As this interviewee suggests, this initial lack of suicide evidence in many regions was a concern from policymakers and could potentially constrain policy development. The interviewee stated that this opened many debates regarding the comprehensiveness of the proposed strategy and the realistic challenges that could be faced during implementation. When asked about scope for improvement, interviewees highlighted the importance of a non-stigmatizing definition of mental health that is consistently adopted across stakeholders and diverse states. A researcher and former member of the technical committee for the National Education Policy development stated:

…we need to clearly define mental health. The more ambiguous it gets, the more ambiguity there is in the evidence (researcher).

### Current gaps in policy implementation in India

Interviewees suggested that a de-stigmatized and consistent definition of mental health will not only increase attention on the political agenda but was thought of as a key contribution to successful implementation and evaluation of policies. The interviewees emphasized that a consistent and increased awareness of mental health will lead to a clearer generation of evidence that can be effectively used to inform policies and interventions with a preventive and promotive aim. In two of the older policies, this blurred and often overlapping roles and responsibilities of multilevel stakeholders can result in low implementation of the policy at the community level.

Adding to this, our analysis suggests that the accountability of all policy evaluation is often not clear, meaning that stakeholders often shift this responsibility to one another. This results in a gap between implementation and evaluation. All of the reviewed documents envisaged that implementation of policies would involve contribution by many multilevel stakeholders. However, several interviewees highlighted there is a lack of a systematic evaluation approach. This was felt to reflect a miscommunication between high-level government officials and ground-level policy actors:

…schools feel very pressured to implement different requirements from the government. They look at any such requirements as extra work which is interfering with their normal work, so it becomes a bit of a burden. This is what I have seen on the ground (mental health practitioner).

Such expectations were also reflected by a government official:

It is the stakeholders who should uphold their duties. They are the beneficiaries, so they should decide what is good and what is not good for them (decision maker).

Furthermore, a school counsellor stated that the potential resistance of schools and teachers in implementing the National Education Policy is not surprising, considering the burden they already feel. On the other hand, an interview with a government official stated the (so far) success of the Education Policy implementation and did not report of any issues, also noting the early stages of the implementation at the time. It is important to highlight that education is a state matter and the implementation of the National Education Policy has been stalled in several states, including Karnataka. Interviewees stated that a lack of resources, limited capacity for implementation and evaluation in schools and the burden placed on teachers can lead to a conflicting perception of how successful current implementation has gone. When asked about scope for improvement, interviewees highlighted the importance of a stronger and regular monitoring system. This was seen as a key mechanism to avoid confusion surrounding implementation responsibility and avoid schools feeling unmanageable burden.

### Involvement of policy actors

Specific roles of policy actors (researchers, policymakers and intermediaries) in generating and using evidence reflect their evidence preferences, relative powers, values and organizational mandates. These roles sometimes influenced the uptake or rejection of certain evidence.

Our analysis identified the following groups of key policy actors who were generally involved in evidence-informed adolescent mental health policymaking: decision makers, intermediaries, researchers and mental health practitioners. The Table 5 below describes the roles of our interviewed participants and their contribution and involvement to each policy. Three of these participants were not involved specifically in the four analysed policies included in this paper but provided detailed insight into the policy and/or evidence processes based on extensive experience and knowledge from previous policies.

**Table 5:** Involvement of study participants across the four policies

	Policies			
Respondent group	National Mental Health Policy (2014)	Ayushman Bharat (2018)	National Education Policy (2020)	National Suicide Prevention Strategy (2022)
Decision makers (MoHFW, MoE and Govt. of Karnataka)		Expert opinion/input involved in the policy development and oversees implementation	Decision maker involved in development of policy and heads the committee for the implementation of the policy	Decision maker involved in agenda-setting through to development of strategy
	Gave oversight regarding policy processes in India and the approaches to evidence uptake, based on experience as a previous government member and child protection specialist			
Intermediaries (regional advisor and media outlets)	Provided insights into previous experience in adolescent mental health policy development. Currently advises the government at central and state level			
				Attended programme launch events, disseminated research to the public
Researchers	Generated evidence that was used to inform policy, and also has experience disseminating to decision makers and development input		Member of expert committee who gave inputs during policy development	Generated evidence that was used to inform agenda-setting and disseminated to decision makers to inform the development of the policy
	Extensive experience generating and disseminating evidence to decision makers. Provided insights into previous child health policy development and input			
	Members of the teams who generated key evidence that informed multiple policies included in this paper. Experience disseminating evidence to decision makers			
Mental health practitioners			Has seen the implementation of the policy at the ground-level across variety of schools	Member of the technical committee for expert opinion and inputs during development

The data from documents and interviews highlighted that government officials from the MoHFW, MoE or Government of Karnataka usually spearheaded development of each policy and had varying levels of power over evidence uptake. This varying power depended on their position within the government and influenced the uptake or rejection of evidence.

Bureaucrats and government officials beyond the health sector were involved in research used to inform the National Suicide Prevention Strategy and the National Mental Health Policy. During an initial research programme, the government expanded both the time period and districts that the project targeted. Interviews with researchers suggested that this would have been difficult to achieve if it was not for the involvement from these bureaucrats and government officials. Several interviewees also suggested that this intersectoral collaboration for research facilitates dissemination from evidence to policy implementation as it sets the policy agenda early on and holds credibility. The interviewee explained that:

It’s my research project but at the same time it’s a programme for them. They said ‘instead of one area, let’s do it in four!’ so then we generate more data so it can convince the government to have larger funding for the entire programme, even after 3 or 4 years (researcher).

Interviewees reported that evidence dissemination to decision makers could be a challenge. Researchers who had generated evidence and subsequently disseminated this evidence to decision makers had first-hand experience in the ways in which government power could directly impact evidence uptake. Several researchers and mental health practitioners were also involved in technical committees or expert groups commissioned by the government during policy development. Document reviews revealed that across all policies, a large number of stakeholders were involved during implementation, many of whom were interviewees in our study.

Interviews with researchers emphasized that the evidence uptake often requires powerful influences, such as media coverage and public pressure, referred to as intermediaries in Table 5, to push evidence into the policy agenda. A researcher involved in the development of the National Suicide Prevention Strategy highlighted that in a previous project launch event, 30 media outlets attended and took ‘bites’ from them, which eventually reached government stakeholders. This was reported as a powerful way to reach all decision makers and encourage discussion and traction amongst high-level stakeholders. Another researcher explained that this can also mean that:

What happens to a research finding to the adaptation to a policy, advocacy note or discourse paper is very challenging…what happens to these findings is just a reflection of the 90% of the findings of the research into one advocacy note…I might take 40% or 50%, based on how I am able to pitch it (researcher).

Although advocacy through media and publishing was generally a positive mechanism to increase evidence dissemination, some researcher interviewees raised concerns about potential for evidence distortion or ‘cherry-picking’.

### Wider involvement and engagement of stakeholders

Majority of interviewees conveyed the importance of understanding community needs and how these should be captured within policies. Interviewees also noted the significance of direct advocacy, such as public engagement activities, to disseminate research findings to the beneficiaries themselves. Researcher interviewees shed light on how the transition from research to policy conversion is often quicker if decision makers are certain that the evidence addresses and appeals to the community needs, greatly influencing their decision to take up that evidence. An interview with a researcher also drew on the importance of involving ‘everyone’, from panchayats to bureaucrats during evidence generation. This was considered by researchers as a mechanism to minimize the gap between research and policy conversion, as if policymakers can be convinced that community needs are addressed then the likelihood of governmental participation increases and can in-turn be used to inform policy agendas. Interviews with researchers reported that key ministries and decision makers such as WHO can be called for consultations for evidence dissemination. A government advisor and researcher stated that it often helps to have the long-term goal in sight and present the policy to decision makers in steps such as short-term, mid-term and long-term steps, as document reviews also portrayed. As an interview with a researcher discussed:

It is not easy for policy makers to accept things which are long-term because it involves a lot of imagination…today’s data is relevant for today’s people (researcher).

Involving young people in decision-making on adolescent mental health policymaking was considered by all respondents across all policies as ‘largely untapped’ and a ‘missed opportunity’ to increase community engagement within policy development and implementation. Interviews with researchers and a government advisor both noted the current gap between evidence generation and policy implementation, the ‘know-do gap’, and the ways in which youth voice engaging in decision-making could be the link between policy agenda and community needs. Holding regular discussions and debates with young people was thought important to generate continuous, dynamic evidence to inform policy that is both contextualized and reflects the ground reality of the beneficiaries, as noted by a government advisor. Interviews with a school counsellor and researchers emphasized that including young people during implementation approaches would also enable real-time input that would likely improve the current lack of evaluation:

It can be done. We have to start thinking about it, believing it and implementing it…it has to happen! Information is something that is only available with the students. Right from primary school, they can learn to be part of decision-making (mental health practitioner).

### Relative powers, values and organizational mandates

The majority of stakeholder interviewees (all researchers, intermediaries and one government official) and in relation to all policies reported a view that actors’ personal preferences and power dynamics impacted evidence uptake. As the quote below describes, during the development of Ayushman Bharat and the National Mental Health Policy, a government stakeholder presented screen addiction data, which was ignored as it was deemed ‘not important’ by a higher-level colleague:

I was personally invested because I thought it was really interesting….it was not added as part of the main curriculum, but we added it as part of the appendix. There were challenges because a lot of us were personally invested in a lot of issues (decision maker).

Notably, two high-level governmental officials were ‘not aware’ of any power dynamics between stakeholders. However, interviews with researchers who disseminate evidence to inform policies reported a view that if a high-level official is not personally invested in particular evidence, it is often rejected. This interviewee did not explicitly state that the evidence was deemed unimportant, but their higher-level colleague was just solely not as invested in it as the interview respondent.

A few respondents also suggested that decision makers will already know which evidence they are using and potentially also the nature of the policy. Interviews with some researchers suggested that sometimes decision makers are more likely to accept evidence that is aligned with their political ideologies and which is specific to only certain members of society, rather than society as a whole. A researcher suggested this is because it is more relatable to the committee group members who may not always represent diverse communities. Respondents stated that this can result in some evidence being overlooked or even promoted into the policy agenda. For example, a researcher interviewee referred to the government using data produced by a large online company due to the potential economic interest associated with this collaboration and evidence input.

These power dynamics influenced evidence uptake between international stakeholders who provide technical expertise or research. Interviews with two researchers who were in the technical committees for the development of two key policies reported that international actors often come with their own organizational agendas, which are likely to be influenced by their funding agencies. One researcher likened these actors to the ‘Tom Cat’ app, alluding to the ways in which the international agencies may just repeat what their funder has asked of them. These interviews also revealed how NGOs often do not fill the gap between data collection, collation and policy formulation leading to think-tanks viewed as more ‘legitimate’ forms of policy input by the government. Similarly, two interviews with government stakeholders noted that sometimes international actors can dominate the policy agenda and that their ideology may be pushed forward or even premeditated to align with political ideals. An interview with a government official shed light on the difficulties this can entail during policy development, and that they often have to endure a lot of ‘give and take’. A specific issue highlighted during the interview is when local actors contextualize or adapt evidence, and international stakeholders view this as not using their evidence.

### Actors’ evidence preferences and likelihood of evidence uptake

Documents showed that most stakeholders preferred formal, quantitative and local evidence and perceived these as robust. However, international data was often used by decision makers due to its perceived credibility and acceptability to the global audience, as stated by researcher interviewees. Interviews noted the lack of in-country mental health data (excluding large-scale surveys such as the National Mental Health Survey and research), which can mean a general uptake of WHO frameworks which are then adapted to local context. Most interviewees agreed that, although international experiences can be helpful, especially from countries and contexts similar to India, localized evidence that reflects the diversity and complexity of mental health in local communities is key. A lack of in-country evidence can increase policy generalizability and as a government advisor reported:

We need local heart and knowledge. Going with the rigid approach that ‘they (international evidence) know everything’ is not always going to work (intermediary).

Similarly, these interviews expressed a need for state and national-level entities to generate data, especially on the social aspects of mental health such as rehabilitation and causation, that can be applied at district-level. A balanced approach to evidence generation, dissemination and uptake can ensure a complementary representation of evidence is used during policy development. It is important to note that both international and local evidence was considered important by all interviewees.

Personal preferences of decision makers can impact evidence dissemination and uptake. Although not reported by government officials, a researcher stated that they either use ‘emotion’ or ‘statistics’ to disseminate research findings to decision makers, depending on who they’re presenting to, for example:

When I am playing to a political leadership I might use the heart, when I’m presenting to a bureaucrat I might go with the facts and numbers which would be more successful (researcher).

The inconsistency of evidence uptake, although based on credibility and quality, is often decided through the ways in which evidence is sold and framed, for example, through advocacy and by ‘convincing’ the policy maker. As reflected by other interviewees, it is important to disseminate evidence to those with a personal interest in the specific policy issue and to even identify those decision makers prior to disseminating the evidence.

## Discussion

This study aims to enhance the understanding of the current role of evidence in four key Indian adolescent mental health policies. Five key issues emerge from our findings. First, all four policies went through stages of agenda setting, development and implementation, though with differences reflecting the nature of each policy issue. However, the uptake of evidence across these policies mostly informed the agenda setting and development stages, and while both informal and formal evidence was used to inform all policies, this varied across policies with greater emphases on formal, quantitative and national-level datasets alongside the international evidence. Second, the context of mental health in India determined evidence-informed policymaking; the stigma surrounding suicide, suicide attempts and mental health disorders, appear to have constrained evidence generation and political prioritization of mental health. Third, relative powers and dynamics of policy actors shaped the uptake of evidence to inform policies, essentially reflecting individual perceptions of robust evidence and organizational agendas. Fourth, our findings reflect the importance of intersectoral collaboration throughout evidence generation and dissemination, as a way to enhance communication between decision-makers and policy beneficiaries to facilitate evidence uptake into policymaking. The use of advocacy and media outlets was reported as a powerful mechanism to enhance this communication.

Below we discuss these issues, followed by identification of implications for improving evidence-informed policymaking, and of study limitations.

### Uptake of different types of evidence across policy processes

The importance of both formal and informal evidence uptake in policy work has been highlighted by Brooks et al. (2023) who emphasized the need to encompass a broader range of evidence and particularly informal evidence [13]. This is especially poignant for mental health policymaking due to the widely documented lack of mental health research, as described by respondents during the National Suicide Prevention Strategy and National Education Policy development [13, 19, 37, 38]. We found that both formal and informal evidence informed mental health policies in India; although, personal experiences and interests were also highly influential over policy content and evidence uptake. Studies from elsewhere highlight that personal experiences and interests were less likely to be reported as ‘rigorous’ or ‘robust’ evidence [5, 6] though were also not as likely to be recognized as informal by high-level stakeholders.

Reflecting on the evidence uptake across different stages of policy processes, evidence was mostly used during the agenda-setting and development stages (Fig. 2) and often comprised formal evidence such as quantitative, national-level survey data or reports. Similarly, WHO frameworks or reports were also used in the development of the National Mental Health Policy, Ayushman Bharat and the National Suicide Prevention Strategy. This is also reflected below as the perceieved most ‘robust’ form of evidence by interviewees. Although formal evidence was largely used in the agenda-setting and development stages of these policies, informal evidence, such expert groups and consultations, were also utilized during these stages. Evidence uptake in policymaking reflects a complex interplay between policy actors and external context, yet all components play a crucial role in influencing the uptake of particular evidence to inform policy decisions. Policy implementation may also take several years to progress, which may explain lack of clear indication of evidence-informed policy implementation as has been seen in the National Education Policy and National Suicide Prevention Strategy [39].

Considering a broad and balanced range of evidence types is critical for evidence-informed mental health policymaking, due to the stigma associated with mental health and the inconsistent definition of mental health. As our findings showed, defining mental health in a consistent way that normalizes the lived experiences and which emphasizes the potential for promoting positive mental health and could reduce stigma. Given the heterogeneity of local understandings of mental health yet the often monocultural definitions of mental health applied in non-Western cultures [41], the complexity of working across cultures and the stigmatization of mental health in these contexts requires both informal and formal evidence to be considered when developing and implementing mental health policies.

In light of this, most respondents preferred formal, local and quantitative evidence that was perceived as ‘robust’. Similarly, other studies reported that maternal health policymakers in Vietnam, India and China preferred local evidence due to the adaptability to the local context, yet often perceived international evidence as high quality and authoritative [6]. The uptake of international frameworks and experiences were also applied in all four policies analysed in this report and can be argued as significantly important when offering lessons for strengthening the role of evidence in mental health agenda-setting in LMICs [13, 40]. It is important to note that local and international evidence can complement each other.

### Contextual influences on evidence-informed health policymaking

Our findings suggest that the context influences evidence-informed mental health policy development, echoing insights from other studies [2, 5, 6, 41]. As highlighted in the adapted framework, the context (in this case stigma and lack of resources) can be seen as an issue cutting across other components of the framework (actors, policy processes and evidence processes) resulting in reduced evidence generation and limited attention on the policy agenda due to competing priorities. Existing research [42, 43] draws attention to the importance of governments and organizations responding to these challenges in the external environment to secure resources and increase awareness of critical policy issues. As mental health is continued to be de-stigmatized within Indian society and although recent efforts to decriminalize suicide through the Mental Healthcare Act have shown, it is important that existing evidence is promoted into the policy agenda.

Similarly, generation of evidence on previously controversial issues, such as suicides and attributed mental health issues, may influence the interests and ideologies of decision-makers. For example, as Gupta and Sagar (2022) address, the Indian public and even legal and health systems may still have limited awareness about mental health issues due to the ambiguity surrounding them [36]. As Kapiriri et al. (2003) and Mosquera et al. (2001) examine, this new generation of evidence can in turn shape resource allocation, decision-making and policy implementation, which in the case of mental health agenda-setting in India, has received limited attention due to competing political priorities such as non-communicable diseases [44–46]. As other reports emphasize, this increased attention to mental health policy and programme in India, a previously neglected sector, is welcomed [47]. However, as the authors suggest, mental health policymaking in India is dominated by a singular discourse that lacks diverse knowledge-practice communities in decision-making, resulting in ‘administration of bio-medical psychiatry at the community-level’ [47].

As our findings show, the approach to policy implementation and often minimal evaluation system is down to both lack of resources and in turn, the burden and responsibility of policy implementation placed on teachers. Gupta and Sagar (2022) [36] outline the various barriers relating to the implementation of several Indian policies, including the National Mental Health Policy (2014) and Mental Healthcare Act (2017). As they discuss, although the National Mental Health Policy aimed to integrate community rehabilitation, it is ground-level implementation is still ‘abysmally low’, undoubtedly due to the lack of resources and limited linkage and involvement of various stakeholders (including training community members in various tasks). Similarly, for better implementation of the National Mental Health Policy, the recent Mental Healthcare Act (2017) should be considered and passed in alignment. Although interviews with a school counsellor and a government stakeholder referred to the responsibility of the National Education Policy implementation, Gupta and Sagar (2022) draw on this in relation to the National Mental Health Policy and that the roles and responsibilities of different stakeholders do not explicitly lead to the successful implementation or tangible change at the ground level [36]. It is also important to note that every state has their own programmes, which are at different levels of implementation. Even national programmes are not implemented at the same time and in same way.

### Relative roles and power dynamics influence evidence processes

The complexities of power dynamics and interplay amongst policy actors have been well-documented, acknowledging the influences that individual and organizational agendas and interests have on evidence uptake [13, 25, 48, 49]. The majority of stakeholders from all policies reported the ways in which powers and positions of high-level actors impacted on which evidence was used to inform each policy. This power affected all steps of evidence processes (generation, dissemination and uptake) and specifically during agenda-setting and policy development stages of the policy process. Of course, different stakeholders suggested different experiences of relationships and dynamics which often reflected their position. For example, two high-level decision-makers explicitly stated that they were ‘not aware’ of any form of power play. In light of this, decision-makers were reportedly more likely to choose evidence that relates to the middle class because majority of committees are middle-class members or evidence that is aligned with the constitution of India. It is important that, as Gore and Parker (2019) discuss, actors at the national level (such as politicians or government officials) do not influence the ‘equity and universality’ of public policy in relation to their own ideologies [49–51].

Likewise, our findings suggest that international stakeholders can sometimes dominate local voices within the policy arena. This can sometimes result in stakeholder views being overlooked, despite their credibility or positive contributions and engagements in policy processes. It is important to continually consider the agendas and interests of all actors involved throughout all policy processes, both international and local, when inputting evidence into contextually complex policies [6]. However, and as interviews with many stakeholders also noted, these international contributions can of course contribute to methodological rigour and pass on valuable experiences.

### Importance of intersectoral collaboration

Stakeholder involvement beyond the health sector during evidence generation was a powerful mechanism for evidence-informed development of all policies. Interviewees stated that this facilitated smoother dissemination to decision makers. It is clear that for successful implementation of mental health programmes in the community, the convergence between various departments such as health, education, welfare, panchayats, etc. can be instrumental. As existing research also reports, the increasing involvement of different policy actors in evidence generation, dissemination and uptake may be a reflection towards participatory policy processes [6, 52]. However, it is important to note that the involvement of non-state actors during evidence dissemination and policy development may also improve evidence uptake. Although our research found the involvement of government counterparts or bureaucrats during evidence generation can be a successful mechanism for improving evidence uptake during dissemination, personal perceptions of ‘robust’ or credible evidence may also influence the uptake of this evidence over other high-quality research or even limit the stakeholders involved in evidence generation in the first place. As a report from European contexts discusses [50], the involvement of these non-state actors can provide useful insights into how public policy is changing and can act as a lens through which to explore policy environments. Similarly, as a paper addressing the Indian context discusses [47], the engagement and learning from diverse initiatives from a myriad of actors may enable a shift from the previously described ‘singular discourse’ and biomedical psychiatry in India.

Similarly, the involvement of media outlets during project launch events were described by an interviewee as a successful method to elevate evidence dissemination to decision-makers and push critical issues onto the policy agenda. This has also been reported in current literature [6], which draws on the use of media outlets and their role, from being an advocate during agenda-setting stages to disseminators of policies in Vietnam. However, sometimes this can lead to the distillation or distortion of key evidence and context, only portraying a specific outline or statistic that can fall into the risk of being ideologically influenced by the organizational agenda of whom is reporting. It is key to actively engage policy makers and contextualize findings of early evidence to ensure a smooth translation from evidence to policymaking, and essentially avoid the misinterpretation of evidence when disseminated into the public sphere. For this to be achieved, guiding standards and active communication with policy makers is encouraged [54]. This will no doubt also help to minimize the current know-do gap that respondents referred to and the challenges of translating scientific evidence into actionable evidence reported in other studies also [53, 54].

The lack of youth voice within these adolescent mental health polices was regarded by all respondents as a significant omission. They view youth contribution as largely untapped despite its potential to provide valuable insights and and linkage from the policy level to community needs. Though the government and high-level officials may decide the extent of agenda-setting and major policy decisions, the involvement of these beneficiaries may indeed be able to steer the process and contribute in ways government officials cannot [55]. Young people could help to shape local and age appropriate definitions of mental health, could identify priorities for youth and could offer innovate policies solutions that would be feasible and effective for their age group.

Adding to this further, our findings also drew attention to the ways in which the direct advocacy to beneficiaries themselves during evidence dissemination can enhance the importance of community needs to decision makers. As reported in Ghana [52], advocacy plays a key role in bringing evidence into the policy-making process and is often needed to communicate this information to citizens and decision makers. Discussed in our findings, the use of advocacy not only portrays the direct benefit to the community or beneficiaries in which it aims to serve, but also as a way to highlight this benefit to the decision maker too.

### Implications for future evidence-informed adolescent mental health policymaking

Our analysis suggests the following implications for enhancing evidence-informed adolescent mental health policymaking in India and beyond. First, different evidence preferences of key stakeholders should be appreciated, while also encouraging use of a balanced representation of evidence throughout the stages of the policy process. Although most stakeholders may prefer formal, quantitative and local evidence, it is important to raise awareness amongst all stakeholders of importance of complementary types of evidence that can be equally useful, for example, informal or unpublished evidence. Similarly, as research [6] also highlights, evidence generation and dissemination should directly prioritize locally-available evidence from reputable actors, including appropriate quantitative and qualitative data sets. Second, it is important for high-level entities to support and engage in generating local-level data that can be applied directly to community needs. Third, our findings highlight the importance of an intersectoral approach to enhance complementary and balanced perspectives in evidence production and policy agendas. An inclusive list of stakeholders can be critical for ensuring participatory policymaking, as well as effectively balancing the often different actor interests, agendas, and relative powers [52]. Fourth, policymakers should recognize and promote the importance of engagement of policy beneficiaries during policy-making, such as youth for adolescent mental health policies. Fifth, mental health can and should be promoted as a key policy priority through the generation of evidence and effective advocacy, to help continue de-stigmatizing mental health in LMICs. It is crucial that a consistent definition of mental health is adapted to tackle associated stigma. This will also ensure more preventive and promotive mental health interventions will be developed, as opposed to the curative services currently proposed. Last, to help minimize the know-do gap that currently exists as a barrier between evidence generation and policy development we call for adequate resources and capacity strengthening for evidence generation, as well as raising awareness of what evidence exists and widely sharing that evidence.

### Study limitations

Although different stakeholder groups were interviewed, 13 interviews is a relatively small sample and may not represent all policy actors. Youth engagement within policymaking was regarded as a missed opportunity and a largely untapped area amongst all respondents. However, no adolescents were interviewed during this research and therefore we cannot assume their interest in engaging in policymaking. However, outcomes from our on-going work in SAMA (forthcoming) indicate that young people want to have a seat at the policy table.

## Conclusions

Adolescent mental health policies are an important policy priority, often facing perceived societal stigma around mental health and competing political priorities. However, increased awareness of critical policy issues can enhance evidence generation for policymaking. Different formal and informal evidence can inform national mental health policies. While most stakeholders prefer formal, quantitative and local evidence, a balanced approach to generation and dissemination of different types of evidence can ensure a complementary representation of evidence. Specific roles of different policy actors in generating and using evidence reflect their evidence preferences, relative powers and values, suggesting the need to consider all these in ensuring a balanced availability of relevant evidence for policymaking. Involvement of government officials within and beyond health sector in research can facilitate evidence-informed policymaking. Engaging the youth or beneficiaries in policymaking can facilitate addressing beneficiary needs and build positive citizenship and youth contributions to society.

## Data Availability

The anonymized dataset generated and analysed during the current study are available from the corresponding author on reasonable request.
